# The mediating role of self-control between physical activity and mobile phone addiction in adolescents: a meta-analytic structural equation modeling approach

**DOI:** 10.3389/fpsyt.2025.1446872

**Published:** 2025-05-19

**Authors:** Hao Lin, Huailong Fan, Qi Fu, Shan Li, Qingzao Liu

**Affiliations:** ^1^ School of Physical Education, Chengdu University, Chengdu, China; ^2^ School of Economics and Management, Shanghai University of Sport, Shanghai, China

**Keywords:** physical activity, mobile phone addiction, self-control, adolescents, meta-analysis, structural equation modeling

## Abstract

**Background and objectives:**

With the rapid advancement of information technology, adolescent cell phone addiction has emerged as a pressing and urgent issue. The combined influence of physical activity (PA) and self-control (SC) on mobile phone addiction (MPA) in adolescents has been investigated in previous studies. However, the strength of the relationship between physical activity and cell phone addiction is not clearly understood. The mediating effect of self-control on this relationship also lacks clarity. Thus, in this study, meta-analytic structural equation modeling methods were employed to evaluate the reliability of effect sizes and the mediating effect of SC.

**Methods:**

Relevant manuscripts from the establishment of databases up to May 2024 were retrieved from five Chinese and English databases: Web of Science, PsycINFO, Pubmed, CNKI (core), and CBM. Meta-analysis was performed using CMA (V3) software, while the Web MASEM application was used to perform path analysis and mediated effects analysis.

**Results:**

This meta-analysis included a total of 48 studies containing 75,541 subjects. The findings of this study revealed that the mean weighted effect size of PA and MPA was -0.204, thereby indicating a low to moderate negative correlation between the two parameters. Meanwhile, the mean weighted effect size of PA and SC was 0.213, which was indicative of a small to medium strength positive correlation between the two. However, SC and MPA were found to share a medium to large strength negative correlation, as revealed by the mean weighted effect size of -0.449. Structural equation model (SEM) results demonstrated that the mediating effect of SC between PA and MPA was -0.091, with the mediating effect having a share of 49.7%.

**Conclusion:**

Adolescent PA and MPA exhibited a low to moderate negative correlation, with SC acting as a partial mediator between the two.

## 1 Introduction

Mobile phones have become an indispensable part of people’s lives with the rapid advancement of technology. While mobile phones offer great convenience, they also lead to a growing dependence and, in many cases, addiction. MPA is an addictive behavior where an individual uses a mobile phone uncontrollably, leading to a series of physiological, psychological, and social problems, including symptoms of withdrawal, tolerance, etc. (1). In fact, MPA is one of the leading non-drug addictions in the 21st century (2). The global summary prevalence of MPA was revealed to be 26.99% as of 2021 by a meta-analytic study (3). However, MPA is more severe in adolescent students. In 2022, a survey revealed that about 36.6% of Chinese college students suffered from MPA (4). MPA can lead to severe consequences for students, including depression (5), anxiety (6), and sleep disorders (7), significantly disrupting their daily lives (8). Therefore, addressing and alleviating MPA is an urgent issue that requires immediate attention.

PA is a healthy habit that reduces the time and frequency of mobile phone use (9). It also enables individuals to exercise their will and strengthen their physical and mental toughness. Interactions with peers in sports foster the development of interpersonal relationships in adolescents. Moreover, the environment of mutual supervision and encouragement helps them to regulate their daily behaviors and conform to the social norms (10), increasing the likelihood of them avoiding MPA-related behaviors. PA can also enhance the dopamine signaling ability of the body, decreasing addiction behavior (11). Consisting of 12 randomized controlled trials, a meta-analysis revealed that PA intervention significantly reduced MPA in adolescents (12). Another study involving Korean adolescents revealed that consistent engagement in physical activities across various intensities effectively prevented MPA. Several other cross-sectional studies have also reported a negative correlation between PA and MPA (13, 14). With advancements in developmental psychology, scholars are increasingly focusing on the mediating mechanisms of PA on MPA. This includes examining variables such as SC, psychological capital, psychological distress, self-esteem, and resilience, etc. A literature review suggests that most studies focused on exploring the indirect impact of SC, a self-regulatory behavior, on MPA. SC is a positive psychological quality that enables individuals to overcome immediate impulses, habits, or automated responses, allowing them to consciously regulate their behavior in alignment with social norms and long-term goals (15). It is considered an important psychological variable.

A study summarizing 17 English-language papers published before 2022. The selected reports discussed the relationship between PA and MPA among adolescents and demonstrated that the two factors shared a moderately strong negative correlation (16). However, that study only included English-language papers and the subjects included in the study were primarily Chinese adolescents, it was essential to search the Chinese-language databases to improve the accuracy of the results. It was also necessary to update the meta-analysis data in a timely manner since dozens of articles were published on related topics in the past two years. Notably, existing meta-analytic studies have concentrated on integrating study effects and exploring possible moderating effect but have not addressed mediating effect. Additionally, the relationship with MPA using PA as a predictor variable and SC as a mediator variable has not yet been explored in any meta-analysis. Thus, this study used meta-analysis to explore the consistencies and discrepancies among existing studies written in both Chinese and English and examine the reported correlations between PA and MPA among adolescents. Furthermore, Structural Equation Modeling was utilized to validate the mediating mechanism of SC. This study aimed to provide a rationale and new perspectives for preventing and intervening in MPA among adolescents.

## 2 Methods

### 2.1 Search strategy

Web of Science, PsycINFO, PubMed, CNKI (core), CBM, and five other Chinese and English databases were searched using AND operator using “physical activit*”/exercise*/”physical fitness”/sport*; addiction/dependenc*/overuse/abuse/”problem use”/”addicted to”; “cell* phone*”/”mobile phone*”/”smart phone*”/”smartphone*” as keywords. The initial search criteria were as follows: literature mentioning quantitative results, details on the relationship between PA and MPA, journal articles, and a search period up to May 2024. The screening resulted in the identification of 657 related journal articles.

**Figure 1:**
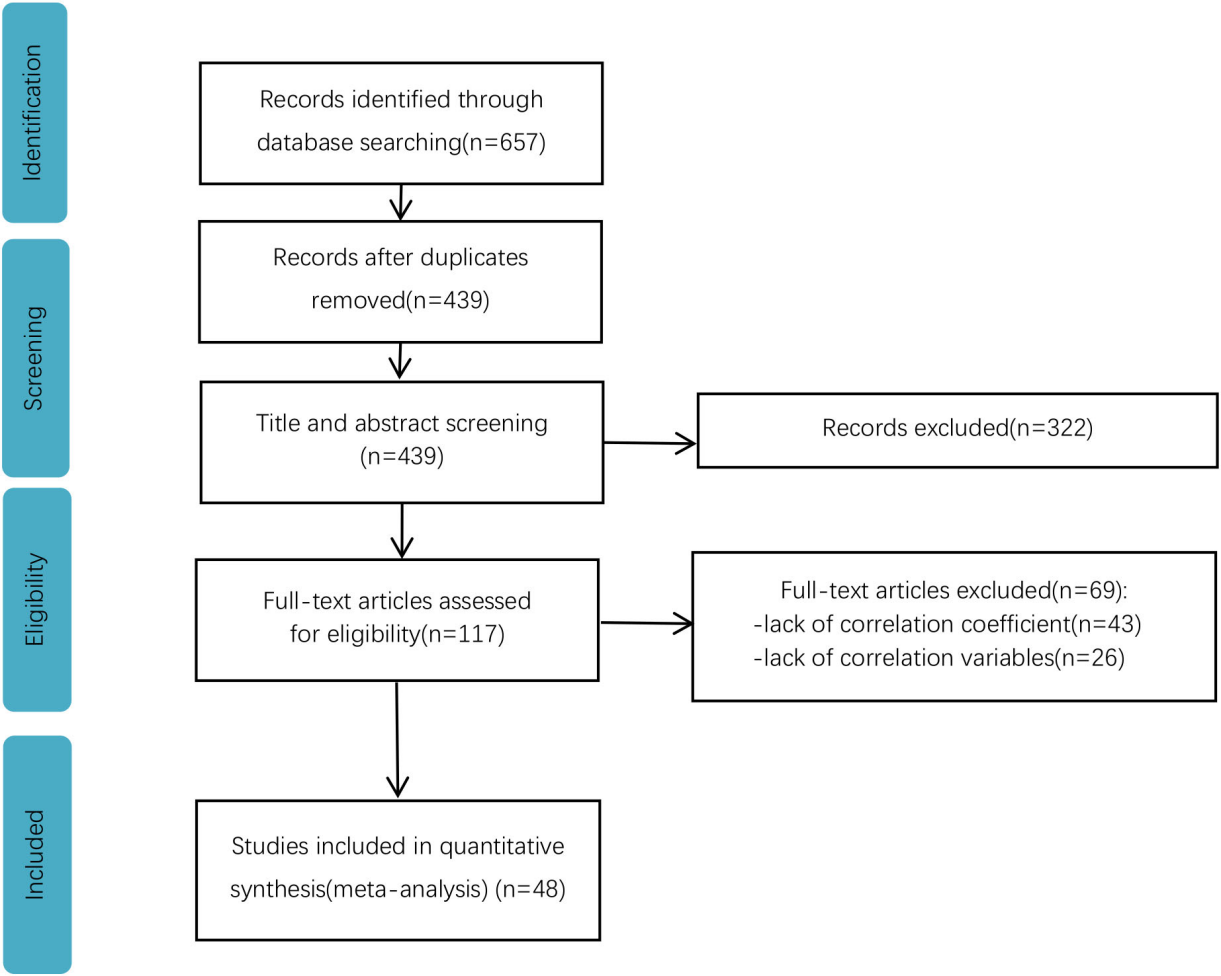
Flowchart of the study selection process.

The selection criteria were as follows: The selected articles were 1) published empirical journal articles; 2) explored the relationship between PA and MPA; 3) provided a correlation coefficient r between PA and MPA; 4) the study sample included a group of adolescents and young adults at the elementary, middle, and collegiate levels; groups involving a particular group of people with clinical physical illnesses or disabilities were excluded; 5) If more than one article included the same data, then the first one published was selected; 6) The Joanna Briggs Institute (JBI) Critical Appraisal Checklist (17) was employed to examine the methodological quality of the included cross-sectional studies, excluding those with a higher risk of bias and lower quality (scores are provided in the Supplementary Material). Finally, 48 papers (8 in Chinese and 40 in English) were obtained. **Figure 1** shows the literature screening.

### 2.2 Data extraction and outcome measures

The coding process comprised two main components: the study description and the statistical analysis of effect values. The study description encompassed details such as the first author and publication year, sample size, population demographics, age, measures of PA, measures MPA, mediating variables, and measurement methods (**Table 1**). The statistical analysis of effect values adhered to specific guidelines: correlation coefficients between PA and MPA were extracted as effect sizes; if the study included SC, its correlation coefficients with PA and MPA, respectively, were also extracted; for longitudinal studies, baseline data were utilized for analysis; if the target variable had multiple dimensions and only individual dimension correlations were reported, the mean correlation was calculated. Both researchers conducted the coding independently, with any disagreements resolved through team discussion involving all members.

**Table 1:** Characteristics of the included studies.

Study	*n*	Age	Country/Region (Population)	PA Measurement	MPA Measurement	Mediator variable (Measurement)
Zhao (18)	2131	12.14 ± 2.29	China (Adolescents)	During the past 7 days, on how many days did you engage in physical activity for a total of at least 30 min per day? (walking, running, playing ball, cycling, sweeping)	SAPS	Self-control (SCS)
Wang et al. (19)	301	20.47 ± 4.99	China (College students)	PARS	MPATS	—
Su et al. (20)	1315	—	China (College students)	In the recent month, how many times per week on average do you engage in physical exercise during your preferred time (daytime or nighttime)? Approximately how long does each exercise session last during your preferred time?	SABAS	—
Kumar et al. (21)	138	19 - 25	India (Youth)	IPAQ	SAS	—
Ke et al. (22)	608	20.27 ± 1.69	China (College students)	PARS	MPATS	Self-esteem (SES)
Jin et al. (23)	930	—	China (College students)	PARS	MPDIS	—
Zhang and Gao (24)	560	16.58 ± 1.00	China (Senior high school student)	PARS	MPPUS	Resilience (Resilience Scale for Chinese adolescents); School Adjustment (School adjustment scale)
Xu et al. (25)	5075	—	China (College students)	PARS	MPATS	—
Wang et al. (26)	1112	21.40 ± 3.20	China (College students)	The frequency of physical activity for a total of at least 60 min weekly	MPAI	—
Wan and Ren (27)	516	12.13 ± 1.95	China (Elementary and Middle school students)	PARS	MPAI	—
Tong and Meng (28)	4399	19.20 ± 2.98	China (College students)	PARS	Mobile Phone Addiction Scale	—
Sezer Efe et al. (14)	437	16.30 ± 1.15	Turkey (Senior high school students)	IPAQ	SAS−SV	—
Niu (29)	514	18.43 ± 0.72	China (College students)	PARS	MPAS	—
Meng and Huang (30)	1933	19.63 ± 1.32	China (College students)	PARS	MPAI	—
Liu and Sun (31)	488	19.21 ± 1.22	China (College students)	PARS	Mobile phone addiction tendency scale	Self-control (SCS)
Li et al. (12)	210	18.7 ± 1.00	China (College students)	IPAQ	SQAPMPU	—
Kim and Ahn (32)	2242	—	South Korea (Elementary and Middle school students)	IPAQ	SAPS	—
Jia (33)	823	18.55 ± 0.83	China (College students)	IPAQ	SAS−SV	—
Han et al. (34)	4959	—	China (College students)	PARS	MPATS	—
Gong et al. (35)	643	19.68 ± 1.40	China (College students)	PARS	SAS	Core self-evaluation (Core Self-Evaluation Scale)
Gao et al. (36)	1019	—	China (College students)	PARS	MPATS	—
Ceylan and Demi̇Rdel (37)	424	19.53 ± 2.13	Turkey (College student)	IPAQ	SAS	—
Cetin et al. (38)	86	18 - 30	Turkey (Youth)	IPAQ	SAS	—
Cao et al. (39)	445	13.86 ± 0.79	China (Middle school students)	PARS	SAS	—
Zhang et al. (40)	649	7 - 24	China (Adolescents)	PARS	Self-assessment questionnaire for adolescents’ mobile phone dependence	Self-Control (SCS)
Zeng et al. (41)	1943	19.75 ± 1.30	China (College students)	PARS	MPATS	Self-Control (SCS); Psychological distress (DASS); Rumination (RRS)
Tong et al. (42)	3609	—	China (College students)	IPAQ	MPATS	—
(43)	9569	16 - 29	China (College students)	IPAQ	MPAI	Subjective well-being (General well-being scale)
Lin et al. (44)	1787	18.85 ± 0.93	China (College students)	IPAQ	SAS	—
Huang et al. (45)	452	23.65 ± 4.13	Chinese Taipei (College students)	IPAQ	SABAS	—
Guo et al. (46)	1433	19.67 ± 1.63	China (College students)	PARS	MPATS	Self-Control (SCS)
Chen et al. (47)	9406	19.58 ± 1.07	China (College students)	IPAQ	MPAI	Psychological Capital (Positive Psychological Capital Questionnaire); Social adaptation (Social Adaptation Diagnostic Questionnaire)
Chao et al. (48)	1575	17 - 27	China (College students)	Weekly exercise frequency	SAS	—
Yang et al. (49)	608	20.06 ± 1.98	China (College students)	PARS	MPATS	—
Ding et al. (50)	1725	19.56 ± 0.95	China (College students)	PARS	MPATS	Self-control (lUSCS-cs)
Tanir (51)	236	—	Pakistan (College students)	IPAQ	SAS-SV	—
Numanoğlu-Akbaş et al. (52)	288	17 - 25	Turkey (College student)	IPAQ	SAS	—
Yang et al. (53)	608	—	China (College students)	PARS	MPATS	Self-Control (SCS)
Haripriya et al. (54)	113	22.15 ± 1.69	India (College student)	IPAQ	SAPS	—
Kim et al. (13)	110	21.03 ± 1.61	South Korea (College students)	3D-Sensor Pedometer	SAPS	—
Wei (55)	1013	—	China (College students)	PARS	MPATS	Self-Control (SCS)
Li et al. (56)	502	18.81 ± 1.66	China (College students)	PARS	MPATS	Psychological distress (Kessler psychological distress scale)
Gong and Yang (57)	1679	19.28 ± 1.28	China (College students)	College students physical exercise questionnaire	Smartphone Addiction Scale for college students	Innovative behavior (Innovative behavior scale); Mental health (SCL-90)
Dong et al. (58)	882	—	China (Junior High School students)	PARS	Mobile phone dependence scale for middle school students	Self-Control (Adolescent Self-control Scale)
Xiao (9)	3122	18.91 ± 1.09	China (College students)	The frequency, duration and intensity of physical activity	MPATS	—
Chen and Zhang (59)	1898	19.31 ± 0.97	China (College students)	IPAQ	MPATS	Interpersonal relation disturbance (ICDS)
Zhen and Ma (60)	418	19.58 ± 1.13	China (College students)	PARS	Adult Smartphone Addiction Scale	—
Yang et al. (61)	608	—	China (College students)	PARS	MPATS	—

n, Sample size; SAPS, Smartphone Addiction Proneness Scale; MPATS, Mobile Phone Addiction Tendency Scale; PARS, Physical Activity Rating Scale; SABAS, Smartphone Addiction Scale Brief Version in Chinese; SAS, 33-item Smartphone Addiction Scale; IPAQ, International Physical Activity Questionnaire; SES, self-esteem scale; MPDIS, Mobile Phone Dependence Index Scale; MPPUS, The twenty-seven-item Mobile Phone Problem Use Scale; MPAI, Mobile phone addiction index; DASS, Depression-Anxiety-Stress Scale; SAS−SV, Smartphone Addiction Scale−Short Version; SCS, Self-Control Scale; SQAPMPU, Self-rating Questionnaire for Adolescent Problematic Mobile Phone Use; DASS, Depression Anxiety Stress Scale; RRS, Ruminative Response Scale; lUSCS-cs, Internet use of Self-control Scale for college students; SCL-90, Symptom Checklist 90; ICDS, Interpersonal Comprehensive Diagnostic Scale.

### 2.3 Data analysis

CMA (V3) software was used to conduct meta-analysis. This study was analyzed using a random effects model (62) since the studies in the individual samples were from different aggregates. The correlation coefficients of individual studies underwent Fisher’s Z transformation before calculating the effect values to exclude the effect of varying sample sizes. The Fisher’s Z transformation formula was as follows:


Fisher's Z=0.5×ln(1+r1−r), Vz=1/(n−3), SEz=Vz,r=(e2z−1)/(e2z+1).

Homogeneity test (*Q*, *I* ^2^), where the Q test follows a χ^2^ distribution, with a significant *Q* value representing the heterogeneity of the effect sizes; *I* ^2^ represents the ratio of the true variance of the effect sizes to the total variance, with > 75% ratio indicating a high degree of heterogeneity among the effect sizes (63).

We employed a mixed-effects model to analyze potential moderating factors, specifically applying random-effects models within subgroups and fixed-effects models between subgroups to test whether the effect size differences across moderator-defined subgroups were statistically significant (64).

The Web MASEM application^1^ developed by Jak and Cheung (65) was used for structural equation modeling path analysis and mediation effects tests.

## 3 Results

This meta-analysis comprised 48 studies, encompassing a total of 75,541 participants aged between 7 and 30 years. Predominantly authored by Chinese scholars, 38 studies were conducted in mainland China, with 39 focusing specifically on college students. The main instruments for measuring physical activity include the International Physical Activity Questionnaire (IPAQ) and the Physical Activity Rating Scale (PARS). MPA was primarily assessed using scales such as the Mobile Phone Addiction Tendency Scale (MPATS), the Smartphone Addiction Proneness Scale (SAPS), and the 33-item Smartphone Addiction Scale (SAS). SC emerged as the most frequently examined mediating variable, alongside psychological capital, psychological distress, self-esteem, psychological resilience, and core self-evaluation.

### 3.1 Homogeneity and publication bias test

As depicted in **Table 2**, the effect values from the chosen articles exhibited significant heterogeneity (p< 0.001), with the proportions of variance attributable to sampling error exceeding 75%. This suggests substantial heterogeneity among the effect sizes, thereby justifying the utilization of a random-effects model.

**Table 2:** Pairwise meta-analysis.

Variable relation	*k*	*r*	95%CI		*z*	*p*	*Q_w_*	*I^2^*	Egger’s (*p*)	Fail-safe *N*
			LL	UL						
PA & MPA	48	-0.204	-0.4257	-0.149	-7.168	0.000	2751.813^***^	98.29%	-1.254 (0.570)	7216
PA & SC	10	0.213	0.163	0.262	8.096	0.000	68.745^***^	86.91%	-2.531 (0.487)	1338
SC & MPA	10	-0.455	-0.520	-0.384	-11.180	0.000	188.826^***^	95.23%	1.220 (0.842)	6713

*k*, The number of effect sizes.

**Figure 2:**
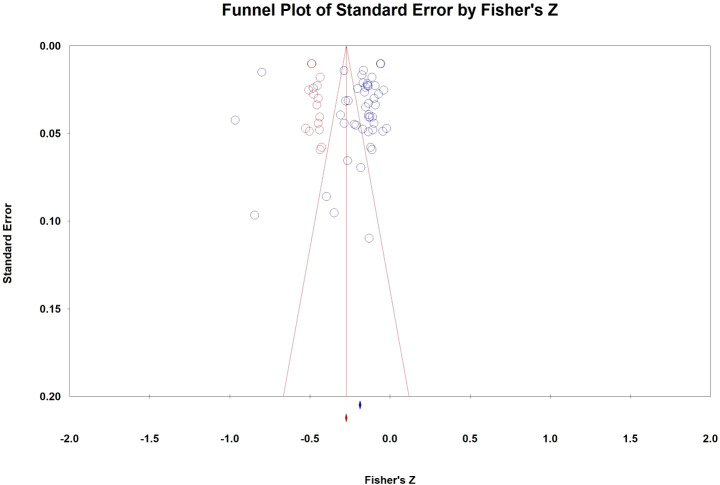
Funnel plots of publication bias with trim and fill.

The publication bias assessment began with an evaluation using a funnel plot. Results indicated that effect values across the three groups were predominantly clustered in the upper section of the funnel plot and were distributed on both sides of the total effect size. This distribution suggested a minimal likelihood of publication bias in the study data. Furthermore, fail-safe numbers for the three groups were calculated as 7,126, 1,338, and 6,713, respectively. These numbers exceeded their respective critical values (5k + 10) of 250, 60, and 60, indicating a robustness against publication bias. Egger’s test for linear regression was then conducted, yielding intercepts of -1.254, -2.531, and 1.220, respectively, with values close to 0. Corresponding p-values of 0.570, 0.487, and 0.842 were all greater than 0.05, suggesting no statistically significant bias. Additionally, employing the trim and fill method revealed that 15 additional studies could be added between PA and MPA to symmetrize the funnel plot. Even after this adjustment, the effect size remained significant at -0.267 (**Figure 2**), indicating relative stability in the results. Overall, these analyses collectively suggested that the publication bias within the meta-analysis was negligible.

### 3.2 Sensitivity analysis

Sensitivity analysis involving alternately excluding individual effect sizes revealed that their removal resulted in only minor variations in the weighted mean r-value (-0.208, -0.188) without altering the overall statistical significance, thereby confirming the robustness and high stability of the findings.

### 3.3 Main effects test

In **Table 2**, the mean weighted effect value of PA versus MPA was -0.204. According to Cohen (66) threshold categorization, where a small correlation is defined as r = 0.10, medium as r = 0.30, and large as r = 0.50, this finding indicates a low to moderate negative correlation between PA and MPA. Additionally, the mean weighted effect value of PA versus SC was 0.213, suggesting a small to medium strength positive correlation. Furthermore, the mean weighted effect value of SC versus MPA was -0.449, indicating a medium to large strength negative correlation between these variables.

### 3.4 Subgroup analysis

Given substantial heterogeneity in effect sizes, we conducted subgroup analyses with educational stage, country/region, and measurement scale as moderators. As shown in **Table 3**, the negative correlation between PA and MPA remained statistically significant across all subgroups. However, only educational stage significantly moderated the PA-MPA relationship (Q = 3.952, df = 1, p = 0.047< 0.05), with a stronger effect size observed in the university subgroup (r = -0.193) compared to the secondary school subgroup (r = -0.115).

**Table 3:** Subgroup analyses of summary correlation between PA and MPA.

Moderator		*Q*	*df*	*p*	*k*	*r*	95%CI		*z*	*p*
							LL	UL		
Educational Stage	1	3.952	1	0.047	37	-0.193	-0.254	-0.131	-6.006	0.000
	2				3	-0.115	-0.161	-0.068	-4.828	0.000
Country/Region	3	0.047	1	0.829	38	-0.200	-0.261	-0.137	-6.157	0.000
	4				10	-0.212	-0.303	-0.117	-4.326	0.000
Scale	5	0.002	1	0.967	13	-0.201	-0.257	-0.143	-6.681	0.000
6	3				-0.198	-0.313		-0.077	-3.179	0.001

1, College; 2, Secondary school; 3, Chinese mainland; 4, Outside of Mainland China; 5, Physical Activity Rating Scale + Mobile Phone Addiction Tendency Scale; 6, International Physical Activity Questionnaire + 33-item Smartphone Addiction Scale; LL, lower limit; UL, upper limit.

### 3.5 Mediation effects test

10 studies were utilized in the mediation effects examination. Employing the theory of meta-analytic Structural equation modeling (MASEM), the mediating effect of SC was assessed through the Web MASEM application. First, the pooled correlation matrix was computed using the Two-Stage Structural Equation Modeling (TSSEM) method proposed by Cheung (67). The results indicated a significant negative correlation between PA and MPA, a significant positive correlation between PA and SC, and a significant negative correlation between SC and MPA, with the findings summarized in **Table 4**.

**Table 4:** Physical activity, mobile phone addiction, and self-control correlation matrix.

Variable	PA	MPA	SC
PA	1.000		
MPA	-0.184	1.000	
SC	0.213	-0.449	1.000

**Figure 3:**
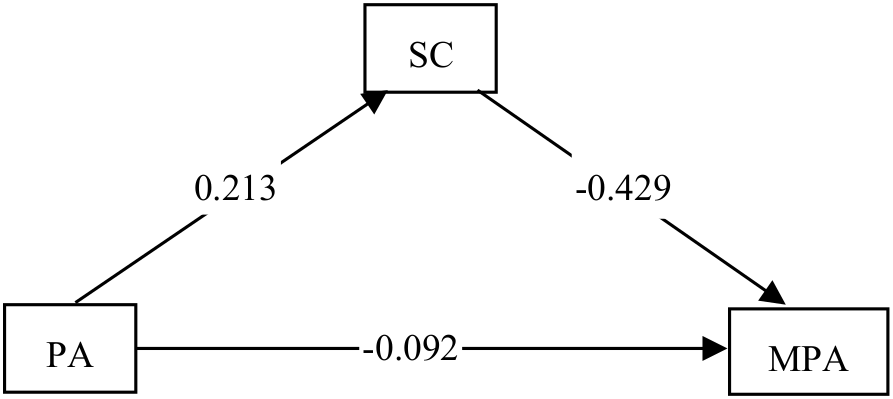
A test of the mediating role of self-control.

In the subsequent step, path analysis was conducted utilizing lavaan syntax^2^ to confirm the SC mediation model (**Figure 3**). The model reached saturation, as indicated by a perfect fit to the data (χ^2^<0.001; df = 0; n=11386; CFI=1; TLI=1; RMSEA=0), suggesting excellent model fit. The results revealed a significant negative direct path from PA to MPA among adolescents, with SC serving as a significant mediator between PA and MPA. This finding elucidates that the effect size of PA on MPA observed in the main effect test appears smaller than its effect size on SC, attributable to the presence of SC as a mediating variable between PA and MPA in adolescents.

As detailed in **Table 5**, PA positively predicted adolescents’ SC (a = 0.213), subsequently influencing MPA (b = -0.429). The indirect effect of PA on MPA was calculated as ab = -0.091, indicating a mediating effect of SC accounting for 49.7% of the total effect (d = -0.183) between PA and MPA. Consequently, approximately 50% of the negative effect of PA on MPA is mediated by SC.

**Table 5:** Path analysis of the mediating effect of self-control.

Mediating variable	*k*	*a*	CI*_a_*	*b*	CI*_b_*	*ab*	CI*_ab_*	*c*	CI*c*	*d*
PA-SC-MPA	10	0.213	0.164, 0.260	-0.429	-0.510, -0.348	-0.091	-0.118, -0.067	-0.092	-0.167, -0.017	-0.183

*k*, The number of effect sizes; CI, Confidence interval; a: the effect of the predictor variable (PA) on the mediator variable (SC); b: the effect of the mediator variable (SC) on the outcome variables (MPA); ab: the indirect effect of the predictor variable (PA) on the outcome variable (MPA) through the mediator variable (SC); c: the direct effect of the predictor variable (PA) on the outcome variable (MPA); d: the total effect of the predictor variable (PA) on the outcome variable (MPA).

## 4 Discussion

This meta-analysis of PA and MPA among adolescents was based on data from 48 publications, which included 48 independent sample studies and 75,541 subjects. Due to the heterogeneous findings, a random effects model was used for analysis. The results indicated the presence of a negative association between adolescents’ PA and MPA, with an effect size of -0.204. These results were consistent with the results of a published meta-analysis article (-0.243) (16). PA has been shown to effectively prevent adolescents from excessive mobile phone usage, reducing their dependency. Some studies have determined that PA can positively predict the satisfaction of students’ basic psychological needs, in turn lowering the likelihood of MPA (68). Embodied cognition theory proposes that PA, by promoting more physically open postures, can mitigate negative emotions and serve as a viable alternative to mobile phone dependence (69). Furthermore, brain science research indicates that PA influences the addiction process by stimulating dopamine release and improving the structure and function of the midbrain dopaminergic system (70). Also, PA stimulates the pituitary gland to release endorphins, enhancing individual pleasure and reducing discomfort in the absence of mobile phones (71). Hence, PA, as a healthy behavior, holds promise for preventing and intervening in MPA. Despite these findings, considerable heterogeneity was observed in the selected studies.

Subgroup analysis revealed statistically significant associations between PA and MPA across all examined subgroups. Notably, educational stage emerged as the sole significant moderator of this relationship, with the correlation magnitude being substantially stronger in the university cohort compared to the secondary school cohort. This observed disparity may be attributed to the heightened academic pressures experienced by secondary school students, particularly those in Mainland China, coupled with stringent parental and teacher-imposed restrictions on smartphone usage - a pattern contrasting sharply with the greater autonomy in device utilization characteristic of university populations. Consequently, the PA-MPA association was attenuated in secondary school students relative to their university counterparts. Geographical factors showed no significant moderating effect, which aligns with the findings reported by Xiao et al. (16). Although no statistically significant differences were observed between subgroups using distinct measurement scales, the potential influence of scale selection on heightened heterogeneity cannot be entirely excluded. The included studies predominantly measured PA and MPA through questionnaire-based assessments or self-reported measures, methodologies inherently associated with elevated measurement error. Such methodological heterogeneity constitutes a substantial contributor to outcome variability in meta-analytic frameworks (72).

Drawing upon the theory of Meta-Analytic Structural Equation Modelling (MASEM), this study delved into the mediating effect of SC as a mediator between adolescents’ PA and MPA, concluding that SC partially mediates the relationship between the two, with the mediated effect representing nearly half of the total effect. Firstly, it was found that PA positively predicts adolescents’ SC, serving as an efficient pathway that bolsters SC. This finding aligns with the strength model of self-control, which posits that SC, akin to a muscle, can be strengthened through appropriate exercise (73). Recent studies on cognitive neuroscience have determined that mild exercise can result in cortical activation in dorsolateral left prefrontal lobe and frontal pole regions, which have been associated with increased SC (74). Additionally, SC was found to be significantly negatively correlated with adolescent mobile phone usage; thus, it acted as a protective factor for adolescent mobile phone use behavior. On the other hand, MPA, which is regarded as a maladaptive behavior, was found to occur in lack of SC and was seen as an impulse control disorder (75). SC is known to be based on executive functioning; consequently, stronger SC can prevent unhealthy mobile phone usage, which, in turn, prevents MPA (76, 77). Based on the self-control theory, SC plays a mediating role and emphasizes that lower levels of SC in individuals might cause delinquent or deviant behaviors, showing a direct causal relationship; the impact of other factors on deviant behaviors could be indirectly realized via the variables of SC (75). PA plays a critical role in indirectly affecting adolescents’ MPA by enhancing their SC. Therefore, in addressing the issue of current college students’ MPA, universities should actively foster a favorable campus sports culture and cultivate students’ regular exercise habits, thereby alleviating their reliance on mobile phone. Meanwhile, parents should actively support middle and primary school students to participate in extracurricular sports activities. Such participation not only reduces screen time after school but also enhances teenagers’ self-control ability, thereby further reducing their tendency to overuse mobile phone.

## 5 Strengths and limitations

Building upon previous research, our study not only explored the relationship between PA and MPA through meta-analysis but also pioneered the application of MASEM to validate the mediating role of SC in the PA-MPA association. Methodologically, this represents an extension of meta-analytic approaches in this research domain. Practically, the findings provide actionable strategies for preventing smartphone addiction among student populations.

However, there are some limitations to this study. 1) Only articles in Chinese and English were included in this study, excluding those in other languages. 2) The high heterogeneity of the included studies reduced the reliability of the findings. 3) The majority of the included studies adopted cross-sectional data. Based on these studies, we are unable to make definite causal inferences. Therefore, longitudinal or experimental research is highly necessary in future studies. 4) Most samples were primarily from China and consisted of college students, with relatively few samples from other geographic regions and groups of primary and secondary school students, which limited the universality of the conclusions.

## 6 Conclusion

In this analysis, the authors collected a substantial number of relevant studies. After examining the relationship between PA and adolescent MPA, the mediating effect of SC between the two was also analyzed using MASEM. Based on the findings, this study concluded that PA and adolescent MPA exhibit a low to medium negative correlation, with SC playing a partial mediating role between the two.

## Data Availability

The original contributions presented in the study are included in the article/**Supplementary Material**. Further inquiries can be directed to the corresponding author.

## Footnotes

1
https://sjak.shinyapps.io/webMASEM/

2 Direct effect test: # Regression coefficients; SC ~ b21*PA; MPA ~ b31*PA + b32*SC; # Variances; PA ~~ 1*PA; SC ~~ p22*SC; MPA ~~ p33*MPA. Indirect effect test: # Regression coefficients; SC ~ beta1*PA; MPA ~ beta2*SC +PA; # Variances; PA ~~ 1*PA; SC ~~ SC;
