# Supplementary material for: The mediating role of self-control between physical activity and mobile phone addiction in adolescents: a meta-analytic structural equation modeling approach
Source: Front Psychiatry. 2025 May 19;16:1446872. doi: 10.3389/fpsyt.2025.1446872 (PMC12127393; doi:10.3389/fpsyt.2025.1446872)
Supplement: Supplementary file 1 [file DataSheet1.pdf]

Supplementary Material

The mediating role of self-control between physical activity and mobile phone addiction in adolescents: A meta-analytic structural equation modeling approach

Hao Lin[1*], Huailong Fan[1], Qi Fu[1], Shan Li[1]， Qingzao Liu[1,2]

*** Correspondence:**

Hao Lin

linhao@cdu.edu.cn

1 Search strategies

1.1 English search strategy (Take the Web of Science for example)

Set#1:TS= (addiction) OR TS=(dependenc*) OR TS=(overuse) OR TS=(abuse) OR TS=("problem use") OR TS=("addicted to")

Set#2:TS= ("cell* phone*") OR TS=("mobile phone*") OR TS=("smart phone*") OR TS=("smartphone*")

Set#3:TS=(exercis*) OR TS= ("physical activit*") OR TS=(sport*) OR TS=("physical fitness")

Set#4:(#1 AND #2 AND #3)

All sets:

Indexes = SCI-EXPANDED, SSCI, CPCI-S, CPCI-SSH.

Timespan=—2024.05.31

Language=English

1.2 Chinese search strategy (CNKI)

Set#1: SU=(手机成瘾 OR 手机依赖)

Set#2: SU= (运动 OR 体育活动 OR 身体活动 OR 锻炼 OR 体力活动)

Set#5: (#1 AND #2)

All sets:

Indexes = 北大核心.

Timespan=—2024.05

Table S1. Research data

Study name	*N*	PA - MPA	PA - SC	SC - MPA
Zhao (2024)	2131	-0.14	0.21	-0.5
Wang et al. (2024)	301	-0.12	—	—
Su et al. (2024)	1315	-0.071	—	—
Kumar et al. (2024)	138	-0.377	—	—
Ke et al. (2024)	608	-0.124	—	—
Jin et al. (2024)	930	-0.133	—	—
Zhang and Gao (2023)	560	-0.747	—	—
Xu et al. (2023)	5075	-0.165	—	—
Wang et al. (2023)	1112	-0.099	—	—
Wan and Ren (2023)	516	-0.278	—	—
Tong and Meng (2023)	4399	-0.664	—	—
Sezer Efe et al. (2023)	437	-0.107	—	—
Niu (2023)	514	-0.1	0.14	-0.57
Meng and Huang (2023)	1933	-0.093	—	—
Liu and Sun (2023)	488	-0.21	0.2	-0.32
Li et al. (2023a)	210	-0.18	—	—
Kim and Ahn (2023)	2242	-0.166	—	—
Jia (2023)	823	-0.151	—	—
Han et al. (2023)	4959	-0.279	—	—
Gong et al. (2023)	643	-0.3	—	—
Gao et al. (2023)	1019	-0.255	—	—
Ceylan and Demi̇Rdel (2023)	424	-0.045	—	—
Cetin et al. (2023)	86	-0.129	—	—
Cao et al. (2023)	445	-0.169	—	—
Zhang et al. (2022)	649	-0.131	0.115	-0.265
Zeng et al. (2022)	1943	-0.14	0.15	-0.45
Tong et al. (2022)	3609	-0.173	—	—
Lu et al. 2022)	9569	-0.058	—	—
Lin et al. (2022a)	1787	-0.153	—	—
Huang et al. (2022)	452	-0.021	—	—

Guo et al. (2022)	1433	-0.158	0.212	-0.607
Chen et al. (2022)	9406	-0.06	—	—
Chao et al. (2022)	1575	-0.04	—	—
Yang et al. (2021)	608	-0.109	—	—
Ding et al. (2021)	1725	-0.445	0.347	-0.315
Tanir (2021)	236	-0.258	—	—
Numanoğlu-Akbaş et al. (2020)	288	-0.112	—	—
Yang et al. (2019)	608	-0.124	0.164	-0.563
Haripriya et al. (2019)	113	-0.335	—	—
Kim et al. (2015)	110	-0.688	—	—
Wei (2023)	1013	-0.27	0.32	-0.46
Li et al. (2023b)	502	-0.22	—	—
Gong and Yang (2023)	1679	-0.2	—	—
Dong et al. (2023)	882	-0.091	0.235	-0.427
Xiao (2022)	3122	-0.112	—	—
Chen and Zhang (2021)	1898	-0.135	—	—
Zhen and Ma (2020)	418	-0.134	—	—
Yang et al. (2020)	608	-0.131	—	—

N, sample size

Table S2. Methodological quality of the studies.

Study	Joanna Briggs Institute appraisal checklist items (0:Out of order；1:Mentioned, but not described in detail；2: Detailed, comprehensive and correct description)										Score	Risk of bias
	1	2	3	4	5	6	7	8	9	10		
Zhao (2024)	2	0	2	2	2	2	2	2	2	2	18	Low
Wang et al. (2024)	2	1	0	1	2	2	2	2	2	2	16	Low
Su et al. (2024)	2	1	0	1	2	2	2	2	2	1	15	Low
Kumar et al. (2024)	1	0	0	1	1	2	2	2	2	1	12	Mid
Ke et al. (2024)	2	2	2	2	1	2	2	2	2	1	18	Low
Jin et al. (2024)	1	0	0	2	2	2	1	2	2	2	14	Low
Zhang and Gao (2023)	2	1	2	2	2	2	0	2	2	2	17	Low
Xu et al. (2023)	2	1	0	2	2	2	0	2	2	1	14	Low
Wang et al. (2023)	1	0	2	2	2	2	2	2	2	1	17	Low
Wan and Ren (2023)	2	0	2	2	2	2	1	2	2	1	16	Low
Tong and Meng (2023)	2	1	1	2	2	2	1	2	2	1	16	Low
Sezer Efe et al. (2023)	2	1	2	2	2	2	2	2	2	2	19	Low
Niu (2023)	1	1	0	1	2	2	1	2	2	2	14	Low
Meng and Huang (2023)	2	0	2	2	2	2	2	2	2	2	18	Low
Liu and Sun (2023)	1	0	1	1	2	2	0	2	2	2	13	Mid
Li et al. (2023a)	1	1	1	2	2	2	2	2	2	2	17	Low
Kim and Ahn (2023)	2	2	0	1	2	2	1	2	2	1	15	Low
Jia (2023)	1	0	2	1	2	2	1	2	2	1	14	Low
Han et al. (2023)	1	1	2	2	2	2	0	2	2	1	15	Low
Gong et al. (2023)	2	0	2	1	2	2	1	2	2	2	16	Low
Gao et al. (2023)	1	1	1	2	2	2	1	2	2	1	15	Low
Ceylan and DemiRdel (2023)	1	0	1	1	2	2	1	2	2	1	13	Mid
Cetin et al. (2023)	2	0	2	2	2	2	2	2	2	1	17	Low
Cao et al. (2023)	2	1	1	1	2	2	0	2	1	1	13	Mid
Zhang et al. (2022)	2	0	1	2	2	2	0	2	2	1	14	Low
Zeng et al. (2022)	1	0	1	2	2	2	1	2	2	2	16	Low
Tong et al. (2022)	1	1	1	2	2	2	0	2	2	1	14	Low
Lu et al. 2022)	2	2	1	2	2	2	1	2	2	1	17	Low
Lin et al. (2022a)	2	0	0	0	2	2	2	2	2	1	13	Mid
Huang et al. (2022)	2	0	2	2	2	2	0	2	2	2	16	Low

Supplementary Material

Guo et al. (2022)	2	2	1	1	2	2	2	2	2	2	18	Low
Chen et al. (2022)	2	2	0	1	2	2	0	2	2	2	15	Low
Chao et al. (2022)	1	1	1	2	2	2	1	2	2	1	15	Low
Yang et al. (2021)	1	2	0	2	2	2	2	2	2	2	17	Low
Ding et al. (2021)	1	0	2	1	2	2	0	2	2	1	13	Mid
Tan (2021)	2	0	0	2	2	2	0	2	2	1	13	Mid
Numanoğlu-Akbaş et al. (2020)	2	0	0	0	2	2	2	2	1	1	15	Low
Yang et al. (2019)	1	2	1	2	2	2	2	2	2	2	18	Low
Haripriya et al. (2019)	2	0	1	1	2	2	2	2	2	1	15	Low
Kim et al. (2015)	1	1	1	1	2	2	2	2	2	1	15	Low
Wei (2023)	2	1	1	2	2	2	0	1	2	1	14	Low
Li et al. (2023b)	1	2	2	2	2	2	0	2	2	2	17	Low
Gong and Yang (2023)	2	0	0	2	2	2	0	2	2	1	13	Mid
Dong et al. (2023)	2	1	1	1	2	2	0	2	2	1	14	Low
Xiao (2022)	1	0	0	2	2	2	1	2	2	1	13	Mid
Chen and Zhang (2021)	2	2	1	2	2	2	0	2	2	1	16	Low
Zhen and Ma (2020)	1	0	0	2	2	2	0	2	2	1	12	Mid
Yang et al. (2020)	1	2	1	2	2	2	1	2	2	1	16	Low

1, Is the purpose of the study clear? Is the foundation of the thesis sufficient? 2, How was the study population selected (Were the study subjects randomly selected, was stratified sampling used to increase sample representation)？ 3, Are inclusion and exclusion criteria clearly described? 4, Does it clearly characterize the sample? 5, Are the data collection tools reliable and valid? (If investigators are used, how about the repeatability of the survey results)？ 6, What are the measures to verify the authenticity of the information? 7, Are ethical issues considered? 8, Is the statistical method correct? 9, Are the statements of the findings appropriate and accurate (are the results and inferences distinguished, and are the results faithful to the data rather than inferences)? 10, Is the importance of the research clearly stated?

frontiers
